# Association of polymorphisms in *C1orf106*, *IL1RN*, and *IL10* with post-induction infliximab trough level in Crohn’s disease patients

**DOI:** 10.1093/gastro/goz056

**Published:** 2019-10-29

**Authors:** Jian Tang, Cai-Bin Zhang, Kun-Sheng Lyu, Zhong-Ming Jin, Shao-Xing Guan, Na You, Min Huang, Xue-Ding Wang, Xiang Gao

**Affiliations:** Department of Gastroenterology, The Sixth Affiliated Hospital, Sun Yat-sen University, Guangzhou, Guangdong, P. R. China; Institute of Clinical Pharmacology, School of Pharmaceutical Sciences, Sun Yat-sen University, Guangzhou, Guangdong, P. R. China; Southern China Center for Statistical Science School of Mathematics, Sun Yat-sen University, Guangzhou, Guangdong, P. R. China; Institute of Clinical Pharmacology, School of Pharmaceutical Sciences, Sun Yat-sen University, Guangzhou, Guangdong, P. R. China; Institute of Clinical Pharmacology, School of Pharmaceutical Sciences, Sun Yat-sen University, Guangzhou, Guangdong, P. R. China; Southern China Center for Statistical Science School of Mathematics, Sun Yat-sen University, Guangzhou, Guangdong, P. R. China; Institute of Clinical Pharmacology, School of Pharmaceutical Sciences, Sun Yat-sen University, Guangzhou, Guangdong, P. R. China; Institute of Clinical Pharmacology, School of Pharmaceutical Sciences, Sun Yat-sen University, Guangzhou, Guangdong, P. R. China; Department of Gastroenterology, The Sixth Affiliated Hospital, Sun Yat-sen University, Guangzhou, Guangdong, P. R. China

**Keywords:** infliximab, inflammatory bowel disease, single nucleotide polymorphism, trough level, multivariate prediction model

## Abstract

**Background:**

Trough levels of the post-induction serum infliximab (IFX) are associated with short-term and long-term responses of Crohn’s disease patients to IFX, but the inter-individual differences are large. We aimed to elucidate whether single gene polymorphisms (SNPs) within *FCGR3A*, *ATG16L1*, *C1orf106*, *OSM*, *OSMR*, *NF-κB1*, *IL1RN*, and *IL10* partially account for these differences and employed a multivariate regression model to predict patients’ post-induction IFX levels.

**Methods:**

The retrospective study included 189 Crohn’s disease patients undergoing IFX therapy. Post-induction IFX levels were measured and 41 tag SNPs within eight genes were genotyped. Associations between SNPs and IFX levels were analysed. Then, a multivariate logistic-regression model was developed to predict whether the patients’ IFX levels achieved the threshold of therapy (3 μg/mL).

**Results:**

Six SNPs (rs7587051, rs143063741, rs442905, rs59457695, rs3213448, and rs3021094) were significantly associated with the post-induction IFX trough level (*P *=* *0.015, *P *<* *0.001, *P *=* *0.046, *P *=* *0.022, *P *=* *0.011, *P *=* *0.013, respectively). A multivariate prediction model of the IFX level was established by baseline albumin (*P *=* *0.002), rs442905 (*P *=* *0.025), rs59457695 (*P *=* *0.049), rs3213448 (*P *=* *0.056), and rs3021094 (*P *=* *0.047). The area under the receiver operating characteristic curve (AUROC) of this prediction model in a representative training dataset was 0.758. This result was verified in a representative testing dataset, with an AUROC of 0.733.

**Conclusions:**

Polymorphisms in *C1orf106*, *IL1RN*, and *IL10* play an important role in the variability of IFX post-induction levels, as indicated in this multivariate prediction model of IFX levels with fair performance.

## Introduction

Infliximab (IFX), a chimeric monoclonal antibody targeting tumor necrosis factor-alpha (TNF-α), has been verified as an effective therapeutic medicine for inflammatory bowel disease (IBD) [1, 2]. Nevertheless, up to one-third of patients primarily show no response to IFX. More than 33% of patients who were initially responsive gradually experienced loss of response [3, 4]. The post-induction serum IFX trough level is not only associated with short-term mucosal healing [5], but also effective in indicating the durable response to IFX [6–8]. Therefore, predicting the post-induction IFX trough level before IFX administration could potentially provide reference for clinical decisions. Although the favorable IFX-level thresholds may vary according to the different treatment goal [9], the widely adopted therapeutic IFX level is ≥3 μg/mL [10–14].

There are large inter-individual differences in IFX trough levels. Many factors may affect IFX clearance, including patient demographics, serum albumin levels, and severity of inflammatory burden [12, 15]. Therefore, it is a challenge to predict before IFX administration whether a patient will achieve this post-induction therapeutic window. The Fc fragment of the IgG receptor IIIa (*FCGR3A*) acts as the receptor of immunoglobulins (IgGs), which could affect the clearance of IFX [16]. The degree of inflammatory burden of IBD patients is influenced by the dysfunction of susceptibility genes for IBD, such as autophagy-related 16 like 1 (*ATG16L1*), chromosome 1 open reading frame 106 (*C1orf106*), oncostatin M (*OSM*), and oncostatin M receptor (*OSMR*) [17–19]. In addition, the nuclear factor kappa B (*NF-κB1*)-induced inflammation pathway is influenced by both interleukin 1 receptor antagonist (IL1RN) and interleukin 10 (IL10), which play a central role in regulating inflammation and could influence the inflammatory burden [20]. The levels of these aforementioned chemokines and cytokines may affect IFX levels by regulating the inflammatory burden. However, whether the single nucleotide polymorphisms (SNPs) within the relevant genes are associated with IFX level, to our knowledge, has not been previously described.

Therefore, we aimed to elucidate whether polymorphisms within *FCGR3A*, *ATG16L1*, *C1orf106*, *OSM*, *OSMR*, *NF-κB1*, *IL1RN*, and *IL10* are associated with IFX levels and to establish a model to predict before IFX administration the likelihood of obtaining the post-induction therapeutic IFX level ≥3 μg/mL.

## Materials and methods

### Patients and data collection

This retrospective study included 189 Crohn’s disease (CD) patients undergoing IFX therapy at the Sixth Affiliated Hospital of Sun Yat-sen University (Guangzhou, China); the patients included in this study were treated between 1 June 2013 and 1 July 2018. All patients were treated with 5 mg/kg of IFX at 0, 2, and 6 weeks during induction therapy. Demographic and clinicopathologic data were recorded, including sex, age, body mass index (BMI), disease duration, disease behavior and location, perianal lesions, previous bowel surgery, combination with thiopurine, serum albumin and hemoglobin level, C-reactive protein (CRP), high sensitivity-CRP (hs-CRP), and erythrocyte sedimentation rate (ESR). This study was approved by the ethics committee of the Sixth Affiliated Hospital of Sun Yat-sen University. All patients provided written informed consent.

### Levels of infliximab

Serum samples were collected at Week 14 to detect trough levels of IFX. Levels of IFX were measured by using an enzyme-linked immunosorbent assay according to the manufacturer’s instructions (Immundiagnostik AG, Bensheim, Germany). An IFX level ≥3 μg/mL was considered to represent the therapeutic level in this study.

### Genotyping

DNA was isolated from EDTA (Ethylene Diamine Tetraacetic Acid) blood samples and extracted by using a TIANamp Genomic DNA Kit (Tiangen Biotech, Beijing, China) according to the manufacturer’s instructions and stored at –80°C until use. A total of 41 SNPs within *FCGR3A*, *ATG16L1*, *C1orf106*, *OSM*, *OSMR*, *NF-κB1*, *IL1RN*, and *IL10* (Supplementary Table 1) were analysed using the MassArray Analyzer system (Sequenom, Inc., San Diego, CA, USA) according to the manufacturer’s instructions. The linkage disequilibrium was calculated using the Haploview bioinformatics software version 4.2 [21] (Broad Institute, Cambridge, MA, USA). A Hardy–Weinberg equilibrium and inherence model was analysed using SNPStats (https://www.snpstats.net/start.htm) [22, 23]. We assessed four inherence models (codominant, dominant, recessive, and overdominant). The inherence model for a specific SNP depends on the Akaike’s information criterion value as well as the *P*-value.

### Definitions of therapeutic outcomes

Simple endoscopic score for CD (SES-CD) values and Harvey–Bradshaw Index (HBI) at baseline and Week 14 were reported by endoscopists. The primary response (PR) at Week 14 was defined as a decrease of >50% from baseline SES-CD or SES-CD ≤2 [24]. The clinical remission (CR) at Week 14 was defined as HBI <5 [25].

### Statistical analysis

Descriptive statistics were provided with median and interquartile range (IQR) or 95% confidence interval (CI) for continuous non-normally distributed variables or with mean and standard deviation for normally distributed data. The Mann–Whitney *U* test (two groups) and the Kruskal–Wallis test (more than two groups) were used to compare continuous non-normality variables and the unpaired *t*-test (two groups) was used to analyse normality variables. The Spearman rank order correlation test was used to evaluate the relationship between continuous variables. Fisher’s exact or Chi-square test was used to analyse discrete variables. All of the above statistical analysis was performed by using SPSS version 24.0 (IBM Corp., Armonk, NY, USA).

The 189 patients were randomly divided into training datasets and testing datasets to develop and verify a prediction model. This process was repeated 100 times to eliminate the randomness in the dataset-splitting process, so we obtained 100 training datasets and 100 testing datasets. Each training dataset was fitted using the Least Absolute Shrinkage and Selection Operator (LASSO) procedure to select variables; this process was also repeated 100 times. Therefore, 10,000 LASSO models were fitted. Then we counted the frequency of those variables included in the models and added those variables into the prediction model according to their frequency from high to low, until the mean area under the receiver operating characteristic curve (AUROC) showed no obvious increase. Thereafter, we choose an AUROC closest to the average for the regression model as a representative split (representative training cohort and representative testing cohort). The receiver operating characteristic (ROC) curve was used to evaluate the performance of the final multivariate model and the optimal threshold prediction value of the multivariate regression model was identified using a maximized Youden’s index. Multivariate regression model analysis was performed using the statistical language R (version 3.3, Foundation for Statistical Computing, Vienna, Austria).

## Results

### Patient characteristics

The relationship between patient characteristics and IFX level at Week 14 is shown in Table 1. All 189 patients were administrated the same dose (5 mg/kg) of IFX at Weeks 0, 2, and 6, and the IFX trough levels were measured at Week 14. The median IFX level was 3.26 μg/mL (IQR, 1.52–5.74 μg/mL). Only 99 patients (52.4%) achieved the IFX therapeutic window (≥3 μg/mL). Sex, age, BMI, disease duration, disease behavior and location, perianal lesions, previous bowel surgery, and thiopurine combination therapy were not significantly associated with the IFX level, whereas higher albumin and hemoglobin levels were significantly associated with increased IFX levels (Table 1). The higher CRP and ESR levels were significantly associated with reduced IFX levels, which suggested that patients with a higher inflammatory burden had an accelerated clearance of IFX (Table 1).

**Table 1.** Associations between patients’ characteristics and IFX levels at Week 14

Demographics and clinical characteristic	Value	Association with 14-week IFX level
Sex		0.167^a^
Male, *n* (%)	141 (74.6)	
Female, *n* (%)	48 (25.4)	
Age, years, median [IQR]	23 [18–28]	0.987^b^
BMI, kg/m^2^, median [IQR]	18.2 [16.6–19.6]	0.069^b^
Disease duration, year, median [IQR]	1 [0.5–4]	0.696^b^
Disease behavior, *n* (%)		0.341^c^
B1	148 (77.9)	
B2	16 (8.4)	
B3	20 (10.5)	
B2 + B3	6 (3.2)	
Disease location, *n* (%)		0.859^c^
L1	16 (8.4)	
L2	6 (3.2)	
L3	145 (76.3)	
L1 + L4	6 (3.2)	
L2 + L4	0 (0)	
L3 + L4	17 (8.9)	
Perianal lesions, *n* (%)	139 (73.2)	0.286^a^
Previous bowel surgery, *n* (%)	32 (16.8)	0.064^a^
Combined with thiopurine, n (%)	98 (51.6)	0.625^a^
Albumin at baseline, g/L, mean ± SD	38.8 ± 5.8	<**0.001**^b^
Hemoglobin at baseline, mg/dL, mean ± SD	112.8 ± 20.2	**0.043** ^b^
hs-CRP at baseline, mg/L, median [IQR]	11.5 [7.2–18.2]	**0.034** ^b^
ESR at baseline, mm/h, median [IQR]	39.0 [24.0–61.0]	**0.002** ^b^
CRP at baseline, mg/L, median [IQR]	17.6 [8.9–35.7]	**0.006** ^b^
CRP at 14th week, mg/L, median [IQR]	3.3 [1.5–5.8]	**<0.001** ^b^

B1, non-stricturing non-penetrating; B2, structuring; B3, penetrating; L1, terminal ileum; L2, colon; L3, ileocolon; L4, upper gastrointestinal; hs-CRP, high sensitive C reaction protein; CRP, C reaction protein; ESR, erythrocyte sedimentation rate.

^a^ Mann–Whitney U test.

^b^ Spearman rank order correlation test.

^c^ Kruskal–Wallis test; those *P*-values <0.05 are highlighted in bold font.

Of the 136 patients with assessed SES-CD values, 119 (87.5%) were responsive to IFX. Compared with patients with a primary non-response to IFX, PR patients had lower ESR levels at baseline (38.5 mm/h [27.0 to 64.0 mm/h] vs 55.0 mm/h [41.0 to 68.5 mm/h], *P *=* *0.038), lower CRP levels at Week 14 (1.0 mg/L [0.5 to 3.8 mg/L] vs 9.4 mg/L [2.9 to 28.4 mg/L], *P *<* *0.001), and higher IFX levels at Week 14 (3.3 μg/mL [1.3 to 6.0 μg/mL] vs 1.7 μg/mL [0.6 to 4.0 μg/mL], *P *=* *0.042) (Table 2). Of the 189 patients with assessed HBI, 180 (95.2%) achieved CR. Compared with patients with a clinical non-remission to IFX, CR patients had lower ESR levels at baseline (38.0 mm/h [24.0 to 60.0 mm/h] vs 56.5 mm/h [42.0 to 70.8 mm/h], *P *=* *0.013), lower CRP levels at baseline (16.6 mg/L [8.3 to 30.8 mg/L] vs 39.2 mg/L [19.4 to 56.8 mg/L], *P *=* *0.036), lower CRP levels at Week 14 (1.0 mg/L [0.5 to 3.7 mg/L] vs 16.8 mg/L [5.1 to 82.4 mg/L], *P *<* *0.001), and higher IFX levels at Week 14 (3.4 μg/mL [1.6 to 5.9 μg/mL] vs 1.1 μg/mL [0.3 to 2.5 μg/mL], *P *=* *0.002) (Supplementary Table 2).

**Table 2.** Relationships between patients’ characteristics and responses

Demographics and clinical characteristic	Primary non-responders (*n* = 17)	Primary responders (*n* = 119)	*P*-value^a^
Sex	–	–	0.377
Male, *n* (%)	11 (64.7)	90 (75.6)	–
Female, *n* (%)	6 (35.3)	29 (24.4)	–
Age, years, median [IQR]	23.0 [15.5–32.0]	23.0 [18.0–28.0]	0.916
BMI, kg/m^2^, median [IQR]	18.6 [16.5–20.4]	18.0 [16.4–19.5]	0.525
Disease duration, years, median [IQR]	2.0 [0.8–3.0]	1.0 [0.5–3.0]	0.468
Disease behavior, *n* (%)	–	–	0.431
B1	13 (76.5)	98 (82.4)	–
B2	1 (5.9)	8 (6.7)	–
B3	3 (17.6)	10 (8.4)	–
B2 + B3	0 (0)	3 (2.5)	–
Disease location, *n* (%)	–	–	0.621
L1	0 (0)	10 (8.4)	–
L2	1 (5.9)	3 (2.5)	–
L3	15 (88.2)	92 (77.3)	–
L1 + L4	0 (0)	2 (1.7)	–
L2 + L4	0 (0)	0 (0)	–
L3 + L4	1 (5.9)	12 (10.1)	
Perianal lesions, *n* (%)	14 (82.4)	91 (76.5)	0.762
Previous bowel surgery, *n* (%)	1 (5.9)	16 (13.4)	0.695
Combined with thiopurine, n (%)	9 (52.9)	69 (59.0)	0.694
Albumin at baseline, g/L, mean ± SD	36.1 [31.7–39.7]	38.4 [34.5–42.1]	0.111
Hemoglobin at baseline, mg/dL, mean ± SD	115.0 [93.0–127.5]	114.0 [95.0–126.4]	0.851
hs-CRP at baseline, mg/L, median [IQR]	12.1 [9.2–33.0]	11.8 [9.5–18.8]	0.512
ESR at baseline, mm/h, median [IQR]	55.0 [41.0–68.5]	38.5 [27.0–64.0]	**0.038**
CRP at baseline, mg/L, median [IQR]	22.6 [7.4–50.2]	20.0 [10.6–38.7]	0.911
CRP at 14th week, mg/L, median [IQR]	9.4 [2.9–28.4]	1.0 [0.5–3.8]	**<0.001**
IFX level at 14th week, μg/mL, median [IQR]	1.7 [0.6–4.0]	3.3 [1.3–6.0]	**0.042**

BMI, body mass index; B1, non-stricturing non-penetrating; B2, structuring; B3, penetrating; L1, terminal ileum; L2, colon; L3, ileocolon; L4, upper gastrointestinal; hs-CRP, high sensitive C reaction protein; IFX, infliximab.

^a^ Chi-square tests or Mann–Whitney U test; those *P*-values <0.05 are highlighted in bold font.

### Genotype distribution and the association of infliximab level

**Figure 1.**
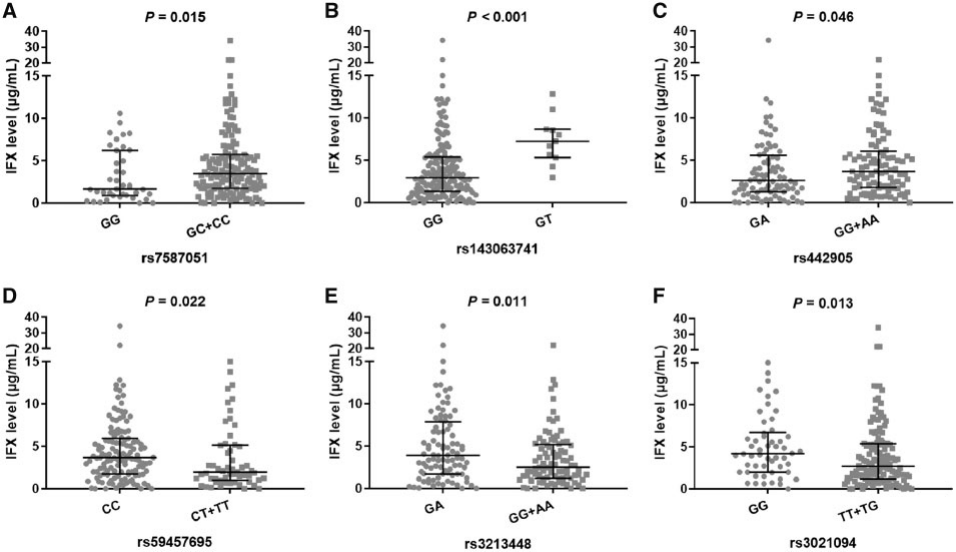
Associations between genotypes and post-induction infliximab trough level. Results of six single-nucleotide polymorphisms (SNP) as shown (A)–(F). Mann–Whitney *U* test.

A total of 41 SNPs within *FCGR3A*, *ATG16L1*, *C1orf106*, *OSM*, *OSMR*, *NF-κB1*, *IL1RN*, and *IL10* were detected. Six SNPs were excluded from analysis because they were not in Hardy–Weinberg equilibrium (Supplementary Table 1). The remaining 35 SNPs were conformed to Hardy–Weinberg expectations. Six SNPs were significantly associated with IFX levels (Figure 1). GG carriers of rs7587051 within *ATG16L1* had lower IFX levels than GC+CC carriers (1.65 μg/mL [95% CI, 0.87–6.22 μg/mL] vs 3.45 μg/mL [95% CI, 1.73–5.74]; *P *=* *0.015, recessive model). The GG genotype of rs143063741 within *ATG16L1* had lower IFX levels than did the GT genotype (2.93 μg/mL [95% CI, 1.31–5.40 μg/mL] vs 7.24 μg/mL [95% CI, 5.32–8.66 μg/mL]; *P *<* *0.001, overdominant model). GA carriers of rs442905 within *C1orf106* showed lower IFX levels than GG+AA carriers (2.59 μg/mL [95% CI, 1.26–5.59 μg/mL] vs 3.68 μg/mL [95% CI, 1.79–6.09 μg/mL]; *P *=* *0.046, overdominant model). Patients with the CC genotype of rs59457695 within *C1orf106* showed significantly higher IFX levels than those of the CT+TT genotype (3.67 μg/mL [95% CI, 1.75–5.95 μg/mL] vs 1.97 μg/mL [95% CI, 1.03–5.15 μg/mL]; *P *=* *0.022, dominant model). GA carriers rs3213448 of *IL1RN* showed higher IFX levels than GG+AA carriers (3.90 μg/mL [95% CI, 1.72–7.88 μg/mL] vs 2.49 μg/mL [95% CI, 1.22–5.19 μg/mL]; *P *=* *0.011, overdominant model). For rs3021094 within *IL10*, the GG genotype showed higher IFX levels than TT+TG (4.18 μg/mL [95% CI, 2.01–6.71 μg/mL] vs 2.68 μg/mL [95% CI: 1.21–5.37 μg/mL]; *P *=* *0.013, dominant model). None of the other 29 SNPs was significantly associated with IFX levels (Supplementary Table 3).

### Multivariate prediction model

**Figure 2.**
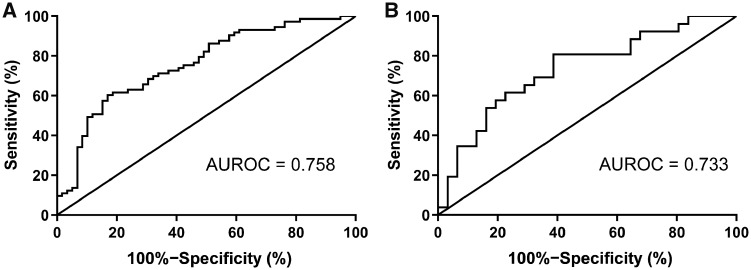
Receiver operating characteristic curve analysis of the performance of the multivariate prediction model in a representative training dataset and in a representative testing dataset. (A) The area under the receiver operating characteristic curve (AUROC) of the multivariate prediction model in a representative training dataset was 0.758 (95% confidence interval [CI], 0.675–0.840, *P *<* *0.001); the sensitivity, specificity, positive predictive value, and negative predictive value were evaluated at 60.3%, 83.1%, 81.5%, and 62.9%, respectively. (B) The AUROC of the multivariate prediction model in a representative testing dataset was 0.733 (95% CI, 0.602–0.865, *P *=* *0.003).

Univariate analysis showed that the level of albumin, CRP, ESR, and the six SNPs identified above were significantly associated with IFX levels. To develop a multivariate prediction model of the patients’ post-induction IFX levels, we divided 189 patients into training (70%) and testing (30%) datasets. Through the LASSO process of variable selection, the following variables were included in the multivariate prediction model: baseline albumin, rs442905, rs59457695, rs3213448, rs3021094 (Table 3). An ROC curve analysis was performed to evaluate the performance of this multivariate prediction model, which revealed that AUROC of this multivariate prediction model in a representative training dataset was 0.758 (95% CI, 0.675–0.840, *P *<* *0.001); the sensitivity, specificity, positive predictive value, and negative predictive value were 60.3%, 83.1%, 81.5%, and 62.9%, respectively. This multivariate prediction model was also verified in a representative testing dataset, with an AUROC of 0.733 (95% CI, 0.602–0.865, *P *=* *0.003), as shown in Figure 2.

**Table 3.** Multivariate logistic-regression analysis

Variable		β	*P*-value	OR	95% CI
Baseline albumin, g/L		0.109	0.002	1.12	1.04–1.20
rs442905	GA vs AA+GG	−0.949	0.025	0.31	0.14–0.68
rs59457695	TT+TC vs CC	−0.865	0.049	0.42	0.17–0.99
rs3213448	GA vs AA+GG	0.761	0.056	2.14	0.99–4.77
rs3021094	TT+TG vs GG	−0.909	0.047	0.40	0.16–0.97

Factors were statistically analysed by multivariate logistic-regression analysis and constant is −2.95.

CI, confidence interval.

### Effects of genotypes on primary responsiveness of IFX, clinical remission to IFX, and 14-week CRP levels

We analysed the effects of six SNPs (rs7587051, rs143063741, rs442905, rs59457695, rs3213448, and rs3021094) on primary responsiveness of IFX, CR to IFX, and 14-week CRP levels. We found no significant association between those SNPs and the PR to IFX (Supplementary Table 4). Interestingly, we found that rs59457695 was significantly associated with CR, whereas rs7587051, rs143063741, rs442905, rs3213448, and rs3021094 were not significantly associated with CR (Supplementary Table 5). Moreover, we found that rs7587051, rs442905, and rs3021094 were weakly associated with 14-week CRP levels (*P *=* *0.089, *P *=* *0.097, *P *=* *0.078, respectively) and rs3213448 was significantly associated with 14-week CRP levels (*P *=* *0.005), but rs143063741 and rs59457695 was not significantly associated with 14-week CRP levels (*P *=* *0.908, *P *=* *0.700, respectively), as shown in Supplementary Table 6.

## Discussion

In this study, to screen for the factors that influence the IFX trough level in CD patients, we analysed the relationship between clinical or genetic indicators and post-induction IFX trough level. Our data showed that albumin, hemoglobin, CRP, and ESR, as well as rs7587051, rs143063741, rs442905, rs59457695, rs3213448, and rs3021094, were significantly associated with IFX trough level. Furthermore, we developed a multivariate regression model based on demographic and pharmacogenetic factors to predict a patient’s post-induction IFX level. AUROCs of this model in training and testing datasets were 0.758 and 0.733, respectively. The sensitivity, specificity, positive predictive value, and negative predictive value were 60.3%, 83.1%, 81.5%, and 62.9%, respectively.

Our data suggested that a higher albumin level was associated with an increased IFX level (*R *=* *0.254, *P *<* *0.001). This finding is consistent with the previously reported result that low albumin could accelerate IFX clearance [15, 26]. This relationship can be partly explained by the common elimination and rescue pathways for both albumin and IgG. Meanwhile, an increased hemoglobin level, as a biomarker of response to IFX [27], was found to be associated with higher IFX levels in this study. Severe inflammation of the mucosa and damage to the intestinal barrier induce luminal protein loss and hypoalbuminemia, resulting in a severe catabolic state and higher IFX clearance [15]. Similar to the results of previous studies, in the present study, we found that the levels of CRP and ESR were negatively associated with IFX levels, which suggests that patients with a higher inflammatory burden have an accelerated clearance of infliximab. In addition, we found that thiopurine combination therapy did not affect the trough level of IFX. This result is in controversy with that of Ruffolo *et al.* [28]. This discrepancy may be explained by the fact that the patients in our study did not undergo thiopurine combination therapy until antibodies appeared.

*ATG16L1*, *C1orf106*, *OSM*, and *OSMR* are IBD-susceptibility genes and genetic polymorphisms in these genes may alter the inflammatory burden and confer on patients an increased risk of IBD [29–31]. *ATG16L1*, a member of the autophagy pathway, is expressed in intestinal epithelial cells and suppresses inflammatory cytokines provoked by the triggers *Nod1* and *Nod2* to relieve inflammation. In our study, the C allele carrier of rs7587051 and T allele carrier of rs143063741 within *ATG16L1* showed higher IFX levels. *C1orf106* is highly expressed in the human intestine and in intestinal epithelial cell lines. Mohanan *et al.* [18] very recently demonstrated that *C1orf106* regulates stability of epithelial adherences junctions by limiting cytohesin-1-dependent ARF6 activation, which plays a key role in regulating the expression of critical adherend junction proteins. Our results indicated that rs442905 and rs59457695 within *C1orf106* were associated with IFX levels. *NF-κB1*, *IL1RN*, and *IL10* are key cytokines involved in immune homeostasis and play important roles in chronic inflammatory conditions. Our study demonstrated that rs3213448 within *IL1RN* and rs3021094 within *IL10* could significantly influence IFX levels. Rs3021094 is located in the first intron of the *IL10* gene. According to the SNPInfo analysis [32], this SNP is predicted to alter a putative transcription factor binding site for SP3, which is the key transcription factor in regulating *IL10* expression. Rs3213448 within *IL1RN* was reported to be associated within IL-1Ra levels [32]; consequently, it would influence the severity of systemic inflammation.

The clinical significance of our present findings is the ability to identify in advance the patients whose post-induction therapeutic IFX levels will be ≥3 μg/mL. Such patients are more likely to have a short-term and durable response to IFX. Therefore, predicting the IFX levels in patients before IFX administration could potentially provide reference for clinical decisions to improve IFX therapeutic effects.

Certain limitations of the present study should be mentioned. First, only 87 patients have 14-week CRP-level data and only the SES-CD values of 136 patients had been assessed at the 14th week. The limited sample size may not provide sufficient statistical power to determine the effect of rs7587051, rs143063741, rs442905, rs59457695, and rs3021094 on the 14-week CRP levels, as well as have resulted in the lack of a statistical association of six SNPs (rs7587051, rs143063741, rs442905, rs59457695, rs3213448, and rs3021094) with the primary responsiveness of IFX. Maybe in a larger sample study, we can find obvious associations between those SNPs and 14-week CRP levels, as well as associations between those SNPs and IFX response, as we have found that rs59457695 was significantly associated with CR in 189 patients. Second, the function of these six SNPs is unknown and the mechanisms of their influence on IFX should be investigated. Third, although we tested the performance of our prediction model in a testing dataset, a prospective study is still needed to validate the discrimination power of this model.

In general, we performed a comprehensive study to analyse the factors that may predict the post-induction trough level before IFX administration. We found that the albumin levels and SNPs of *Clorf106*, *IL1RN*, and *IL10* play key roles and can be used to predict the therapeutic level of IFX with a fair performance. This finding may provide reference for making clinical decisions and improve therapeutic effects after further validation in large clinical studies.

## Supplementary data

Supplementary data is available at *Gastroenterology Report* online.

## Authors’ contributions

J.T. and C.B.Z. contributed to the study design, research performance, sample collection, acquisition of data, and manuscript writing; K.S.L. and N.Y. contributed to study design, data analysis, and revision of the draft; Z.M.J. and S.X.G. participated in the experimental process, data collection, and revision of the draft; M.H. contributed to the study design, data analysis, and revision of the draft; X.D.W. and X.G., as the co-corresponding authors, were involved in the study design and revision of the draft. All authors read and approved the final version of this paper.

## Funding

This study was funded by grants from the National Natural Science Foundation of China [Grant No. 81573507]; the National Natural Science Foundation of China [Grant No. 81473283]; the National Natural Science Foundation of China [Grant No. 81173131]; the National Natural Science Foundation of China [Grant No. 81320108027]; the Natural Major Projects for Science and Technology Development from Science and Technology Ministry of China [Grant No. 2012ZX09506001-004]; and the Major Scientific and Technological Project of Guangdong Province, China [Grant No. 2011A080300001].

## Supplementary Material

goaa056_supplementary_data
