# Supplementary material for: Association of polymorphisms in C1orf106, IL1RN, and IL10 with post-induction infliximab trough level in Crohn’s disease patients
Source: Gastroenterol Rep (Oxf). 2019 Oct 29;8(5):367–73. doi: 10.1093/gastro/goz056 (PMC7603865; doi:10.1093/gastro/goz056)
Supplement: goaa056_supplementary_data [file goaa056_supplementary_data.zip › 2019-047 Supplement Table 1.docx]

**2019-047 Association of polymorphisms in *C1orf106*, *IL1RN*, *IL10* with** **postinduction** **infliximab** **trough level in** **Crohn’s disease patients**

Jian Tang^1,^**^#^**, Cai-Bin Zhang^2,^**^#^**, Kun-Sheng Lyu^3^, Zhong-Ming Jin^2^, Shao-Xing Guan^2^, Na You^3^, Min Huang^2^, Xue-Ding Wang^2,^**^*^**, Xiang Gao^1,^

**Supplement tables**

**Supplement Table 1.** SNP characteristics

| Gene | Rs number | Most severe consequence **^a^** | MAF | Hardy-Weinberg equilibrium | Linkage SNP |
| --- | --- | --- | --- | --- | --- |
| *FCGR3A* | rs112142198 | Intron | 0.040 | 0.25 | rs67775399 |
|  | rs61801820 | Intron | 0.019 | 1 | No |
|  | rs117758842 | Intron | 0.260 | 0.08 | No |
|  | rs10917571 | Intron | 0.323 | **<0.01** | No |
|  | rs114010589 | Missense | 0.413 | **<0.01** | No |
|  | rs111504845 | Synonymous | 0.471 | 0.11 | rs200030468, rs200244242, rs79418205 |
|  | rs56150752 | Intron | 0.035 | **<0.01** | rs6687275, rs74387071, rs56095771, rs12131312, rs7539036, rs139755165, rs79090924, rs12131862, rs373184583 |
|  | rs67295569 | Intron | 0.176 | **<0.01** | rs78154415, rs143596860, rs140299449, rs61803026, rs35674644, rs144577943 |
|  | rs35276103 | Intron | 0.414 | 0.44 | rs72702132, rs146653557, rs148885671, rs78603229, rs111384507, rs74127051, rs34540988, rs111340912, rs35519204, rs112363639, rs74127055, rs111974271, rs72702116, rs72702111, rs112045543, rs72702109, rs34380170, rs187814751, rs74127074, rs79158401, rs35139848, rs143989790, rs74538320 |
| *ATG16L1* | rs7587051 | Intron | 0.453 | 1.00 | No |
|  | rs12616785 | Intron | 0.169 | 0.61 | rs78765795 |
|  | rs143063741 | Intron | 0.029 | 1.00 | No |
|  | rs74660875 | Intron | 0.097 | 0.23 | No |
|  | rs13032115 | Intron | 0.156 | 1.00 | rs35944010, rs2341566 |
|  | rs4663396 | Intron | 0.159 | 0.27 | rs7595856 |
|  | rs56805998 | Intron | 0.324 | 0.40 | rs13021297, rs13021302 |
|  | rs7587633 | Intron | 0.494 | 0.35 | rs10929033, rs2289475, rs1045095, rs6861, rs10929323, rs11894529, rs7563345, rs11685932, rs7574945, rs6431259, rs4663136, rs1816753, rs1596016, rs35812841, rs3792110, rs930449, rs6431262, rs2241876, rs3749079, rs2119504, rs7595748, rs2241877, rs6737398, rs6431655, rs6431659 |
| *C1orf106* | rs61740234 | Missense | 0.137 | 0.35 | No |
|  | rs442905 | Intron | 0.454 | 0.30 | rs374827 |
|  | rs59457695 | Upstream variant 2 KB | 0.159 | 0.43 | No |
|  | rs59757713 | Intron | 0.165 | 0.29 | rs2275487, rs78710594, rs199712902 |
| *OSM* | rs75951407 | Intron | 0.098 | 0.69 | No |
| *OSMR* | rs357287 | Intron | 0.233 | 1.00 | No |
|  | rs595740 | Intron | 0.309 | 0.39 | rs168568 |
|  | rs13357358 | Intron | 0.005 | 1.00 | rs10472311, rs1393110, rs61166819, rs1430420 |
|  | rs834006 | Intron | 0.052 | **0.05** | rs56092427, rs1270710, rs1239347 |
|  | rs78776521 | Intron | 0.040 | 0.25 | rs375665881, rs3805563, rs9791140, rs114215450, rs16867829, rs148724036, rs77867511, rs74767211, rs78255653 |
|  | rs115277096 | Intron | 0.458 | 0.47 | rs7709749, rs10051319, rs7703639, rs184373356, rs142090164, rs148834777, rs3805565, rs6865980, rs6862959, rs61333312, rs139176105, rs75481125, rs10051334, rs112480830, rs3805561, rs370028418, rs13188094, rs985106, rs9790908 |
| *NF-kB1* | rs4648141 | Intron | 0.069 | 0.60 | No |
|  | rs7674004 | Downstream gene variant | 0.497 | 0.46 | rs11726195 |
| *IL1RN* | rs315926 | Intron | 0.230 | 1.00 | No |
|  | rs315949 | Downstream variant | 0.108 | **0.01** | No |
|  | rs396201 | Downstream variant 500B | 0.302 | 0.6 | rs397211, rs9005, rs3087270 |
|  | rs315933 | Downstream variant | 0.427 | 0.77 | rs315930, rs315927, rs1630153, rs315931 |
|  | rs77853995 | Intron | 0.051 | 0.39 | rs72950891 |
|  | rs1688072 | Intron | 0.066 | 1.00 | No |
|  | rs117929702 | Intron | 0.083 | 0.36 | No |
|  | rs1665190 | Intron | 0.372 | 0.88 | rs1794066, rs1665192, rs439154 |
|  | rs55709272 | Intron | 0.927 | 1.00 | rs28648961, rs13382561, rs62158854, rs10171849, rs17207494, rs7580634, rs62158853 |
|  | rs3213448 | Intron | 0.435 | 0.55 | rs4251991, rs4252001, rs380092, rs4251993, rs2071459, rs928940, rs3087262, rs3181052 |
| *IL10* | rs3021094 | Intron | 0.479 | 0.47 | No |

MAF: Minor Allele Frequency.

**^a^**From Ensembl Genome Browser databases (<http://www.ensembl.org/index.html>); These *P* values ≤ 0.05 were highlighted in bold font. SNP, single nucleotide polymorphism; MAF, minor allele frequency.
