# Supplementary material for: Association of polymorphisms in C1orf106, IL1RN, and IL10 with post-induction infliximab trough level in Crohn’s disease patients
Source: Gastroenterol Rep (Oxf). 2019 Oct 29;8(5):367–73. doi: 10.1093/gastro/goz056 (PMC7603865; doi:10.1093/gastro/goz056)
Supplement: goaa056_supplementary_data [file goaa056_supplementary_data.zip › 2019-047 Supplement Table 2.docx]

**2019-047 Association of polymorphisms in *C1orf106*, *IL1RN*, *IL10* with** **postinduction** **infliximab** **trough level in** **Crohn’s disease patients**

Jian Tang^1,^**^#^**, Cai-Bin Zhang^2,^**^#^**, Kun-Sheng Lyu^3^, Zhong-Ming Jin^2^, Shao-Xing Guan^2^, Na You^3^, Min Huang^2^, Xue-Ding Wang^2,^**^*^**, Xiang Gao^1,^

**Supplement tables**

**Supplement Table 2.** Relationships between patient's characteristics and clinical remission

| Demographics and clinical characteristic | Clinical non-remission patients  (*n* = 9) | Clinical remission patients  (*n* = 180) | *P* value **^a^** |
| --- | --- | --- | --- |
| Sex | – | – | 0.695 |
| Male, *n* (%) | 6 (66.7) | 135 (75.0) | – |
| Female, *n* (%) | 3 (33.3) | 45 (25.0) | – |
| Age, years, median [IQR] | 23.0 [14.0-36.5] | 23.0 [18.3-28.0] | 0.845 |
| BMI, kg/m^2^, median [IQR] | 18.6 [15.3-20.1] | 18.2 [16.6-19.7] | 0.857 |
| Disease duration, years, median [IQR] | 2.0 [0.7-6.5] | 1.0 [0.5-3.0] | 0.477 |
| Disease behavior, *n* (%) | – | – | 0.556 |
| B1 | 7 (77.8) | 140 (77.8) | – |
| B2 | 0 (0) | 16 (8.9) | – |
| B3 | 2 (22.2) | 18 (10.0) | – |
| B2 + B3 | 0 (0) | 6 (3.3) | – |
| Disease location, *n* (%) | – | – | 1.000 |
| L1 | 0 (0) | 16 (8.8) | – |
| L2 | 0 (0) | 5 (2.8) | – |
| L3 | 8 (88.9) | 137 (76.1) | – |
| L1 + L4 | 0 (0) | 6 (3.3) | – |
| L2 + L4 | 0 (0) | 0 (0) | – |
| L3 + L4 | 1 (11.1) | 16 (8.9) | – |
| Perianal lesions, *n* (%) | 8 (88.9) | 131 (72.8) | 0.449 |
| Previous bowel surgery, *n* (%) | 0 (0) | 32 (17.8) | 0.361 |
| Combined with thiopurine, *n* (%) | 3 (33.3) | 95 (52.8) | 0.317 |
| Albumin at baseline, g/L, mean ± SD | 36.1 [30.8-39.5] | 38.8 [35.1-42.5] | 0.051 |
| Hemoglobin at baseline, mg/dL, mean ± SD | 107.0 [89.0-124.1] | 115.0 [96.0-127.0] | 0.144 |
| hs-CRP at baseline, mg/L, median [IQR] | 11.7 [9.2-29.5] | 11.5 [6.3-16.9] | 0.559 |
| ESR at baseline, mm/h, median [IQR] | 56.5 [42.0-70.8] | 38.0 [24.0-60.0] | **0.013** |
| CRP at baseline, mg/L, median [IQR] | 39.2 [19.4-56.8] | 16.6 [8.3-30.8] | **0.036** |
| CRP at 14^th^ week, mg/L, median [IQR] | 16.8 [5.1-82.4] | 1.0 [0.5-3.7] | **< 0.001** |
| IFX level at 14^th^ week, μg/mL, median [IQR] | 1.1 [0.3-2.5] | 3.4 [1.6-5.9] | **0.002** |

BMI: body mass index; B1: nonstricturing nonpenetrating; B2: structuring; B3: penetrating; L1: terminal ileum; L2: colon; L3: ileocolon; L4: upper gastrointestinal；hs-CRP: high sensitive C reaction protein; IFX: infliximab;

**^a^**Chi-Square Tests or Mann-Whitney U-test. These *P*-values < 0.05 were highlighted in bold font.
