# Supplementary material for: Association of polymorphisms in C1orf106, IL1RN, and IL10 with post-induction infliximab trough level in Crohn’s disease patients
Source: Gastroenterol Rep (Oxf). 2019 Oct 29;8(5):367–73. doi: 10.1093/gastro/goz056 (PMC7603865; doi:10.1093/gastro/goz056)
Supplement: goaa056_supplementary_data [file goaa056_supplementary_data.zip › 2019-047 Supplement Table 3.docx]

**2019-047 Association of polymorphisms in *C1orf106*, *IL1RN*, *IL10* with** **postinduction** **infliximab** **trough level in** **Crohn’s disease patients**

Jian Tang^1,^**^#^**, Cai-Bin Zhang^2,^**^#^**, Kun-Sheng Lyu^3^, Zhong-Ming Jin^2^, Shao-Xing Guan^2^, Na You^3^, Min Huang^2^, Xue-Ding Wang^2,^**^*^**, Xiang Gao^1,^

**Supplement tables**

**Supplement Table 3.** Genotype distribution and association of infliximab (IFX) level

| Gene | Rs number | Genotype | No. of patients | Median and IQR of IFX level μg/mL | *P* value **^a^** | Inherence model |
| --- | --- | --- | --- | --- | --- | --- |
| *FCGR3A* | rs112142198 | AA | 174 | 3.26 [1.32-5.67] | 0.364 | NA **^b^** |
|  |  | AT | 14 | 4.21 [1.94-8.48] |  |  |
|  | rs61801820 | CC | 179 | 3.30 [1.55-5.95] | 0.238 | NA **^b^** |
|  |  | CG | 7 | 2.00 [0.33-4.21] |  |  |
|  | rs117758842 | CC | 105 | 3.05 [1.53-5.68] | 0.865 | Dominant |
|  |  | CT+TT | 78 | 3.33 [1.62-5.76] |  |  |
|  | rs111504845 | GA | 105 | 4.07 [1.25-6.61] | 0.166 | Overdominant |
|  |  | GG+AA | 83 | 2.55 [1.51-5.12] |  |  |
|  | rs35276103 | CC | 59 | 3.70 [1.73-6.79] | 0.177 | Dominant |
|  |  | CT+TT | 122 | 2.93 [1.16-5.61] |  |  |
| *ATG16L1* | rs12616785 | TT | 4 | 1.34 [0.15-5.78] | 0.220 | Recessive |
|  |  | CC+CT | 185 | 3.29 [1.53-5.70] |  |  |
|  | rs74660875 | AA | 149 | 3.07 [1.60-5.69] | 0.578 | NA **^b^** |
|  |  | AC | 36 | 3.42 [1.33-6.14] |  |  |
|  | rs13032115 | AG | 50 | 3.79 [1.54-6.94] | 0.324 | Overdominant |
|  |  | AA+GG | 136 | 2.93 [1.51-5.53] |  |  |
|  | rs4663396 | CC | 134 | 3.39 [1.26-5.69] | 0.766 | Dominant |
|  |  | CT+TT | 52 | 3.03 [1.67-7.24] |  |  |
|  | rs56805998 | AA | 22 | 2.40 [0.88-6.51] | 0.568 | Recessive |
|  |  | GG+GA | 163 | 3.35 [1.60-6.79] |  |  |
|  | rs7587633 | CC | 45 | 3.49 [1.78-5.52] | 0.381 | Dominant |
|  |  | CT+TT | 119 | 2.99 [1.06-5.83] |  |  |
| *C1orf106* | rs61740234 | CC | 140 | 3.25 [1.27-5.72] | 0.381 | Dominant |
|  |  | CT+TT | 46 | 4.02 [1.68-8.02] |  |  |
|  | rs59757713 | AA | 7 | 5.07 [0.74-5.50] | 0.730 | Recessive |
|  |  | GG+GA | 181 | 3.25 [1.53-6.77] |  |  |
| *OSM* | rs75951407 | CC | 154 | 3.39 [1.58-5.81] | 0.283 | Dominant |
|  |  | CT+TT | 35 | 2.11 [1.20-5.51] |  |  |
| *OSMR* | rs357287 | AA | 111 | 3.36 [1.54-6.02] | 0.495 | Dominant |
|  |  | AC+CC | 78 | 2.83 [1.32-5.49] |  |  |
|  | rs595740 | GA | 85 | 3.54 [1.63-6.77] | 0.126 | Overdominant |
|  |  | GG+AA | 101 | 2.75 [1.22-5.45] |  |  |
|  | rs13357358 | TT | 187 | 3.25 [1.51-5.65] | 0.403 | NA **^b^** |
|  |  | TA | 2 | NA **^c^** |  |  |
|  | rs78776521 | CC | 1 | NA **^c^** | 0.443 | Recessive |
|  |  | GG+GC | 185 | 3.29 [1.51-5.77] |  |  |
|  | rs115277096 | TG | 100 | 3.56 [1.70-5.91] | 0.191 | Overdominant |
|  |  | TT+GG | 89 | 2.52 [1.03-5.69] |  |  |
| *NF-kB1* | rs4648141 | AA | 1 | No **^a^** | 0.678 | Recessive |
|  |  | GG+GA | 188 | 3.27 [1.52-5.79] |  |  |
|  | rs7674004 | AA | 48 | 3.43 [1.08-6.09] | 0.922 | Recessive |
|  |  | GG+GA | 136 | 3.03 [1.52-5.95] |  |  |
| *IL1RN* | rs315926 | CC | 112 | 2.92 [1.26-5.55] | 0.117 | Dominant |
|  |  | CT+TT | 77 | 3.65 [1.69-6.96] |  |  |
|  | rs396201 | CC | 19 | 2.00 [1.69-4.46] | 0.263 | Recessive |
|  |  | TT+TC | 170 | 3.65 [1.74-7.74] |  |  |
|  | rs315933 | TC | 89 | 3.25 [1.63-6.03] | 0.554 | Overdominant |
|  |  | TT+CC | 97 | 3.29 [1.51-5.50] |  |  |
|  | rs77853995 | GG | 1 | No **^a^** | 0.873 | Recessive |
|  |  | AA+AG | 184 | 3.30 [1.52-5.95] |  |  |
|  | rs1688072 | AA | 164 | 3.16 [1.51-5.91] | 0.915 | NA **^b^** |
|  |  | AG | 25 | 3.52 [1.59-5.14] |  |  |
|  | rs117929702 | GG | 2 | No **^a^** | 0.392 | Recessive |
|  |  | AA+AG | 184 | 3.30 [1.51-5.95] |  |  |
|  | rs1665190 | TT | 26 | 3.60 [1.25-5.28] | 0.903 | Recessive |
|  |  | CC+CT | 158 | 3.25 [1.52-6.06] |  |  |
|  | rs55709272 | CC | 1 | No **^a^** | 0.494 | Recessive |
|  |  | TT+TC | 185 | 3.30 [1.58-5.87] |  |  |

**^a^**Mann-Whitney *U* test; **^b^**There are only two genotypes in this polymorphism, which is not enough to determine its inherence model; **^c^**When the number of patients is <3, no mean and interquartile range (IQR).
