# Supplementary material for: Association of polymorphisms in C1orf106, IL1RN, and IL10 with post-induction infliximab trough level in Crohn’s disease patients
Source: Gastroenterol Rep (Oxf). 2019 Oct 29;8(5):367–73. doi: 10.1093/gastro/goz056 (PMC7603865; doi:10.1093/gastro/goz056)
Supplement: goaa056_supplementary_data [file goaa056_supplementary_data.zip › 2019-047 Supplement Table 4.docx]

**2019-047 Association of polymorphisms in *C1orf106*, *IL1RN*, *IL10* with** **postinduction** **infliximab** **trough level in** **Crohn’s disease patients**

Jian Tang^1,^**^#^**, Cai-Bin Zhang^2,^**^#^**, Kun-Sheng Lyu^3^, Zhong-Ming Jin^2^, Shao-Xing Guan^2^, Na You^3^, Min Huang^2^, Xue-Ding Wang^2,^**^*^**, Xiang Gao^1,^

**Supplement tables**

**Supplement Table 4.** Genotypes and primarily response to infliximab

| rs number | Genotype | *P* value **^a^** | OR | 95% CI |
| --- | --- | --- | --- | --- |
| rs7587051 | GC + CC VS GG | 0.118 | 0.41 | 0.14-1.25 |
| rs143063741 | GT VS GG | 0.595 | 0.94 | 0.90-0.98 |
| rs442905 | GG+AA VS GA | 0.124 | 2.48 | 0.76-8.16 |
| rs59457695 | CT+TT VS CC | 0.179 | 0.42 | 0.11-1.54 |
| rs3213448 | GG+GA VS AA | 0.655 | 0.79 | 0.28-2.22 |
| rs3021094 | TT+TG VS GG | 0.569 | 1.41 | 0.43-4.62 |

**^a^**Chi-Square Tests. OR, odds ratio; CI, confidential interval.
