# Supplementary material for: Association of polymorphisms in C1orf106, IL1RN, and IL10 with post-induction infliximab trough level in Crohn’s disease patients
Source: Gastroenterol Rep (Oxf). 2019 Oct 29;8(5):367–73. doi: 10.1093/gastro/goz056 (PMC7603865; doi:10.1093/gastro/goz056)
Supplement: goaa056_supplementary_data [file goaa056_supplementary_data.zip › 2019-047 Supplement Table 5.docx]

**2019-047 Association of polymorphisms in *C1orf106*, *IL1RN*, *IL10* with** **postinduction** **infliximab** **trough level in** **Crohn’s disease patients**

Jian Tang^1,^**^#^**, Cai-Bin Zhang^2,^**^#^**, Kun-Sheng Lyu^3^, Zhong-Ming Jin^2^, Shao-Xing Guan^2^, Na You^3^, Min Huang^2^, Xue-Ding Wang^2,^**^*^**, Xiang Gao^1,^

**Supplement tables**

**Supplement Table 5.** Genotypes and clinical remission to infliximab

| rs number | Genotype | *P* value **^a^** | OR | 95% CI |
| --- | --- | --- | --- | --- |
| rs7587051 | GC + CC VS GG | 0.433 | 1.70 | 0.42-6.91 |
| rs143063741 | GT VS GG | 1.000 | 1.07 | 1.03-1.11 |
| rs442905 | GG+AA VS GA | 1.000 | 1.21 | 0.29-5.00 |
| rs59457695 | CT+TT VS CC | 0.034 | 1.47 | 1.33-1.63 |
| rs3213448 | GG+GA VS AA | 1.000 | 1.06 | 0.30-3.79 |
| rs3021094 | TT+TG VS GG | 0.727 | 0.61 | 0.13-2.97 |

**^a^**Chi-Square Tests. OR, odds ratio; CI, confidential interval.
