# Supplementary material for: Association of polymorphisms in C1orf106, IL1RN, and IL10 with post-induction infliximab trough level in Crohn’s disease patients
Source: Gastroenterol Rep (Oxf). 2019 Oct 29;8(5):367–73. doi: 10.1093/gastro/goz056 (PMC7603865; doi:10.1093/gastro/goz056)
Supplement: goaa056_supplementary_data [file goaa056_supplementary_data.zip › 2019-047 Supplement Table 6.docx]

**2019-047 Association of polymorphisms in *C1orf106*, *IL1RN*, *IL10* with** **postinduction** **infliximab** **trough level in** **Crohn’s disease patients**

Jian Tang^1,^**^#^**, Cai-Bin Zhang^2,^**^#^**, Kun-Sheng Lyu^3^, Zhong-Ming Jin^2^, Shao-Xing Guan^2^, Na You^3^, Min Huang^2^, Xue-Ding Wang^2,^**^*^**, Xiang Gao^1,^

**Supplement tables**

**Supplement Table 6.** Genotypes and CRP level at 14-week

| rs number | Genotype | CRP level at 14-week  mg/L median [IQR] | *P* value **^a^** |
| --- | --- | --- | --- |
| rs7587051 | GC + CC | 1.8 [1.0-5.9] | 0.089 |
|  | GG | 5.2 [1.0-17.8] |  |
| rs143063741 | GT | 3.7 [1.0-7.1] | 0.908 |
|  | GG | 2.5 [1.0-8.2] |  |
| rs442905 | GG+AA | 1.6 [0.8-6.2] | 0.097 |
|  | GA | 3.7 [1.2-9.8] |  |
| rs59457695 | CT+TT | 1.8 [0.9-8.6] | 0.700 |
|  | CC | 2.8 [1.1-8.2] |  |
| rs3213448 | GG+GA | 3.7 [1.2-9.6] | 0.005 |
|  | AA | 1.4 [0.7-4.1] |  |
| rs3021094 | TT+TG | 2.8 [1.2-8.7] | 0.078 |
|  | GG | 1.1 [0.8-6.0] |  |

**^a^**Mann-Whitney *U*-test. CRP, C-reactive protein; IQR, interquartile range.
